# Identifying a Shared Metabolomic Signature in Mother–Newborn Dyads With and Without Gestational Diabetes: Implications for Early Metabolic Programming

**DOI:** 10.3390/ijms27219786

**Published:** 2026-10-31

**Authors:** Isabel Omaña-Guzmán, Juan Carlos López-Alvarenga, Berenice Palacios-González, Fernanda Jaramillo-Pimentel, Luis Lanz-Pérez, Amhed Alvarado-González, Cristina Díaz-Olivares, José Alberto Carranco-Martínez, Marcela Vela-Amieva, Isabel Ibarra-González, Nayely Garibay-Nieto

**Affiliations:** 1 Pediatric Obesity Clinic and Wellness Unit, Hospital General de México “Dr. Eduardo Liceaga”, Mexico City 06720, Mexico; nutricion.isa@gmail.com (I.O.-G.); fernandajaramillo_@outlook.es (F.J.-P.); luislanz19@gmail.com (L.L.-P.); amhed437@gmail.com (A.A.-G.); cdiaz2471@gmail.com (C.D.-O.); 2 Division of Population Health & Biostatistics, School of Medicine, University of Texas Rio Grande Valley, Edinburg, TX 78539, USA; juan.lopezalvarenga@utrgv.edu; 3 Laboratorio de Genómica del Envejecimiento del INMEGEN en el Centro de Investigación sobre Envejecimiento (CIE-CINVESTAV Sede Sur), Mexico City 14330, Mexico; bpalacios@inmegen.gob.mx; 4 Departamento de Ginecología y Obstetricia, Hospital General de México “Dr. Eduardo Liceaga”, Mexico City 06720, Mexico; jacarrancomx@yahoo.com.mx; 5 Laboratorio de Errores Innatos del Metabolismo y Tamiz, Instituto Nacional de Pediatría, Mexico City 04530, Mexico; mvela@pediatria.gob.mx; 6 Unidad de Genética de la Nutrición, Instituto de Investigaciones Biomédicas, Universidad Nacional Autónoma de México (UNAM), Mexico City 04510, Mexico; icig@iibiomedicas.unam.mx

**Keywords:** gestational diabetes, metabolomics, metabolic programming, amino acids, acylcarnitines

## Abstract

Pregnancy is a critical window for metabolic programming, during which adverse intrauterine exposures may influence long-term health. GDM alters the intrauterine environment, but its role in early metabolic programming remains poorly understood. Thus, the objective of this study was to identify the shared metabolomic signature in mother–newborn dyads and to assess whether GDM modifies this signature. For this purpose, we conducted a cross-sectional study including mother–newborn dyads recruited at the General Hospital of Mexico. Blood samples were collected from dyads. Amino acids and acylcarnitines were quantified by tandem mass spectrometry. Sparse partial least squares (sPLS) analysis was used to identify shared metabolomic signatures within mother–newborn dyads. Linear mixed-effects models evaluated the modifying effect of GDM on shared metabolites. A total of 112 mother–newborn dyads (68 without GDM and 44 with GDM) were included. Two shared metabolomic signatures were identified. The first, characterized by arginine, methionine, acetylcarnitine, and free carnitine, showed a strong maternal–newborn correlation. Although this core signature was preserved in both groups, valine and alanine contributed exclusively to the GDM-exposed dyads. In conclusion, a shared metabolomic signature related to fetal development and energy metabolism was conserved regardless of GDM status, although GDM partially modified this signature. These findings provide insights into metabolic processes potentially involved in early metabolic programming.

## 1. Introduction

Pregnancy is a critical developmental stage during which an adverse intrauterine environment can permanently alter fetal physiology and metabolism, thereby increasing susceptibility to chronic diseases across the life course [1].

Gestational diabetes (GDM) is defined as any degree of carbohydrate intolerance first recognized during pregnancy, resulting from a combination of increased insulin resistance and inadequate compensatory insulin secretion due to pancreatic β-cell dysfunction [2,3]. It is one of the most common pregnancy complications, with a global prevalence of approximately 14% in 2021 according to the International Association of Diabetes in Pregnancy Study Group’s criteria [4]. GDM alters the intrauterine environment through maternal hyperglycemia and associated metabolic and inflammatory disturbances, impairing placental function and nutrient transport to the fetus, thereby influencing fetal growth and development [2].

Epidemiological studies have observed that GDM is associated with short-, medium-, and long-term adverse outcomes for both mother and offspring. In offspring, it increases the risk of macrosomia and large-for-gestational-age birth, and in the long term is associated with an increased risk of obesity, type 2 diabetes mellitus (T2DM), and cardiovascular disease [2,5,6]. However, the biological mechanisms underlying these associations are not fully understood. In this context, metabolomics provides a comprehensive characterization of metabolic phenotypes and offers a powerful approach to investigate the biological mechanisms underlying early metabolic programming [7]. Nevertheless, most metabolomic studies have evaluated the effects of GDM on maternal and neonatal metabolomic signatures separately, rather than considering the mother–newborn dyad as an integrated biological system. Given the close metabolic interplay between mother and fetus mediated by the placenta, investigating the metabolome of the mother–newborn dyad may provide a more comprehensive understanding of the metabolic mechanisms underlying fetal programming [8].

Few studies have integrated maternal and neonatal metabolomic data, and to the best of our knowledge, none have identified a shared metabolomic signature in mother–newborn dyads or evaluated whether GDM modifies this shared signature. Characterizing these shared metabolic signatures may provide novel insights into the mechanisms linking maternal metabolism with neonatal metabolism and fetal metabolic programming. Because the neonatal metabolome reflects the combined influence of maternal metabolism, placental function, and the newborn’s metabolic adaptation to extrauterine perinatal life, studying the mother–newborn dyad during this critical period provides a unique opportunity to investigate the mechanisms underlying early metabolic programming. Therefore, the objective of this study was to identify the shared metabolomic signature in mother–newborn dyads and to assess whether GDM modifies the shared metabolomic signature.

## 2. Results

### 2.1. Characteristics of Mother–Newborn Dyads

A total of 112 mother–newborn dyads (68 non-GDM and 44 GDM) were included. Maternal and neonatal characteristics according to GDM status are summarized in Table 1. Compared with mothers without GDM, those with GDM were older and had a higher pre-pregnancy body mass index (BMI), and higher fasting glucose, triglyceride, and HbA1c concentrations. Neonatal anthropometric characteristics did not differ significantly between groups.

Regarding treatment among mothers with GDM, 50.0% (n = 22) received metformin monotherapy, 9.1% (n = 4) received insulin monotherapy, 18.2% (n = 8) received combined therapy with metformin and insulin, and 22.7% (n = 10) were managed with lifestyle interventions alone.

Absolute maternal and neonatal metabolite concentrations are presented in Supplementary Table S1. Significant differences in maternal concentrations of alanine, methionine, tyrosine, acetylcarnitine (C2), propionylcarnitine (C3), butyrylcarnitine (C4), dodecenoylcarnitine (C12:1), and oleoylcarnitine (C18:1) were observed between the non-GDM and GDM groups. However, none of these differences remained statistically significant after adjustment for multiple comparisons using the Benjamini–Hochberg FDR procedure. Regarding neonatal metabolite concentrations, significant differences were observed in valine, dodecanoylcarnitine (C12), and 3-hydroxyhexadecanoylcarnitine (C16-OH) between groups. After FDR adjustment, only the difference in valine concentrations remained statistically significant. Additionally, scatter plots of paired maternal and neonatal concentrations of metabolites contributing to the shared metabolomic signatures, stratified by GDM status, are presented in Supplementary Figure S1 to illustrate their distribution and within-dyad relationships.

### 2.2. Shared Metabolomic Signatures Within Mother–Newborn Dyads

**Figure 1:**
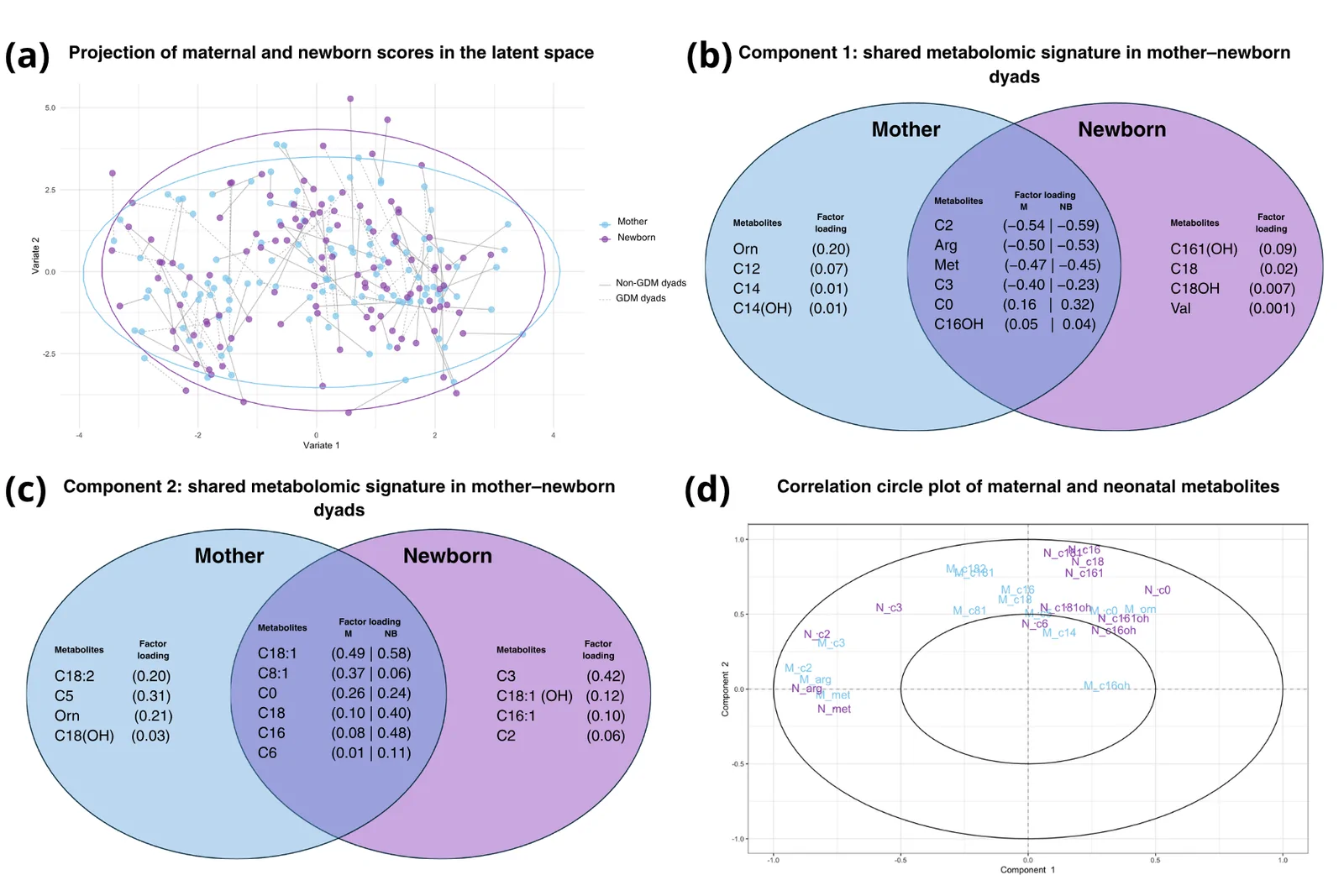
Identification of the shared metabolomic signature in mother–newborn dyads by sPLS. (**a**) Projection of maternal and newborn scores onto Components 1 and 2 in the sPLS latent space. Purple circles represent newborns, and blue circles represent their mothers. Solid lines connect mother–newborn pairs in non-GDM dyads, whereas dotted lines connect mother–newborn pairs in GDM dyads. (**b**) Metabolites comprising the shared metabolomic signature of Component 1 (acetylcarnitine [C2], arginine, methionine, propionylcarnitine [C3], free carnitine, and 3-hydroxyhexadecanoylcarnitine [C16-OH]), as well as the metabolites uniquely identified in mothers and newborns, together with their corresponding factor loadings. (**c**) Metabolites comprising the shared metabolomic signature of Component 2 (long-chain acylcarnitines, C0 and C6). (**d**) Correlation circle showing the association of maternal and neonatal metabolites with Components 1 and 2. Component 1 was primarily characterized by amino acids, free carnitine, and short-chain acylcarnitines, whereas Component 2 was mainly characterized by long-chain acylcarnitines. Purple (N) indicates newborn metabolites, whereas blue (M) indicates maternal metabolites.

Derived from sparse Partial Least Squares (sPLS), we retained two components with positive Q^2^ (Component 1: Q^2^ = 0.066; Component 2: Q^2^ = 0.043). Component 1 showed a strong co-structure between maternal and newborn metabolomic profiles (r = 0.895). Figure 1a shows the shared latent space between maternal and neonatal scores which evidences this co-structure and the coordinated metabolic signature within mother–newborn dyads.

The metabolites comprising the shared metabolomic signature of Component 1 were C2, arginine, methionine, C3, free carnitine (C0), and C16-OH, all exhibiting concordant loading directions. This shared metabolomic signature is presented in Figure 1b, together with the metabolites uniquely identified in mothers and newborns by the sPLS analysis.

Component 2 showed a moderate co-structure between maternal and neonatal metabolomic profiles (r = 0.399 between maternal and neonatal Component 2 scores). The shared metabolites in this component were long-chain acylcarnitines, C0 and hexanoylcarnitine (C6), all with positive and concordant loading directions. As is shown in Figure 1d, the correlation circle revealed that Component 1 was primarily characterized by amino acids and short-chain acylcarnitines, whereas Component 2 was mainly characterized by long-chain acylcarnitines.

### 2.3. Effect of Gestational Diabetes on Shared Metabolomic Signatures

**Figure 2:**
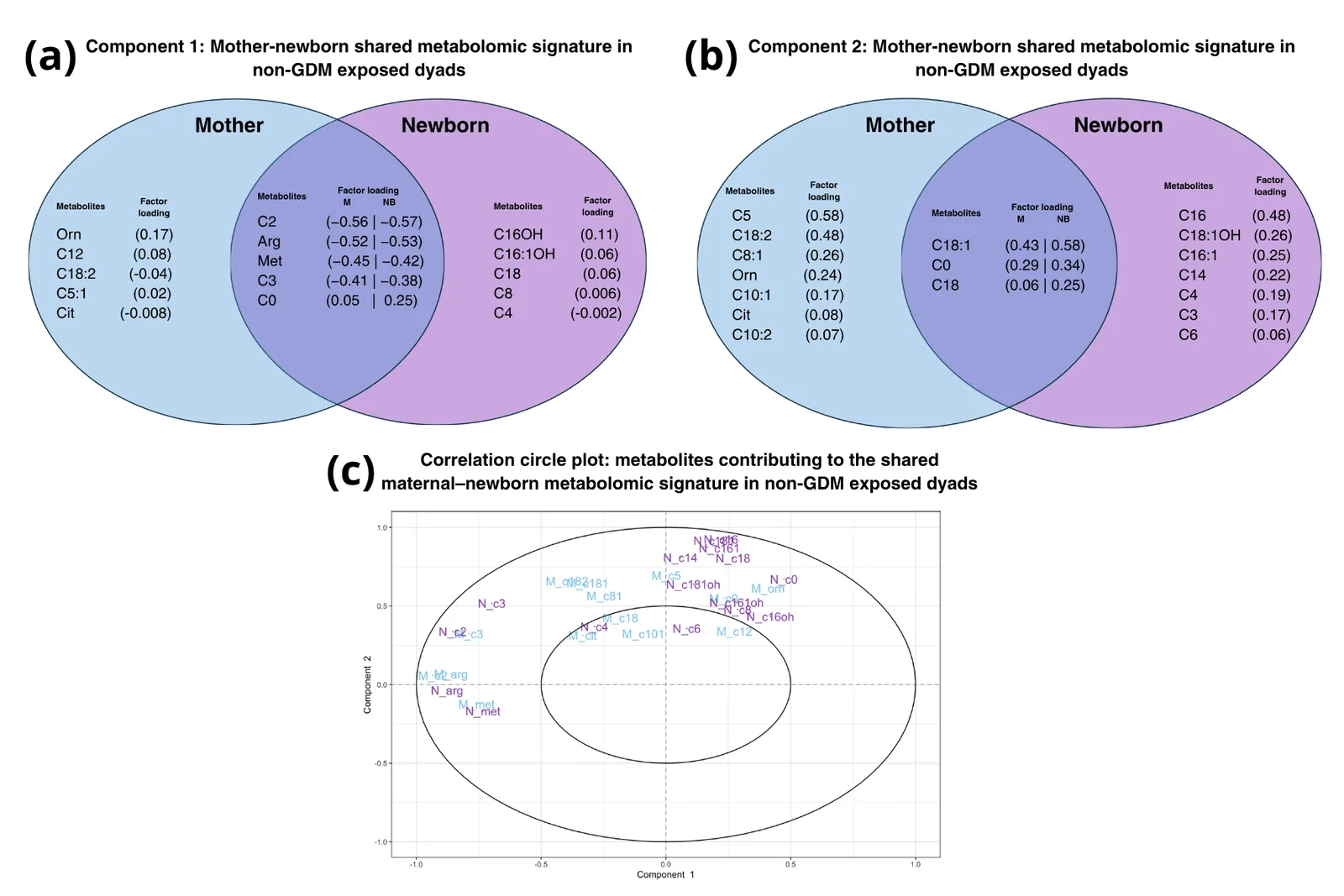
Identification of the shared metabolomic signature in non-GDM-exposed mother–newborn dyads using sPLS. (**a**) Metabolites comprising the shared metabolomic signature of Component 1 in non-GDM mother–newborn dyads, together with their corresponding factor loadings. (**b**) Metabolites comprising the shared metabolomic signature of Component 2 in non-GDM mother–newborn dyads, together with their corresponding factor loadings. (**c**) Correlation circle showing the association of maternal and neonatal metabolites with Components 1 and 2 in non-GDM mother–newborn dyads. Purple (N) indicates newborn metabolites, whereas blue (M) indicates maternal metabolites.

**Figure 3:**
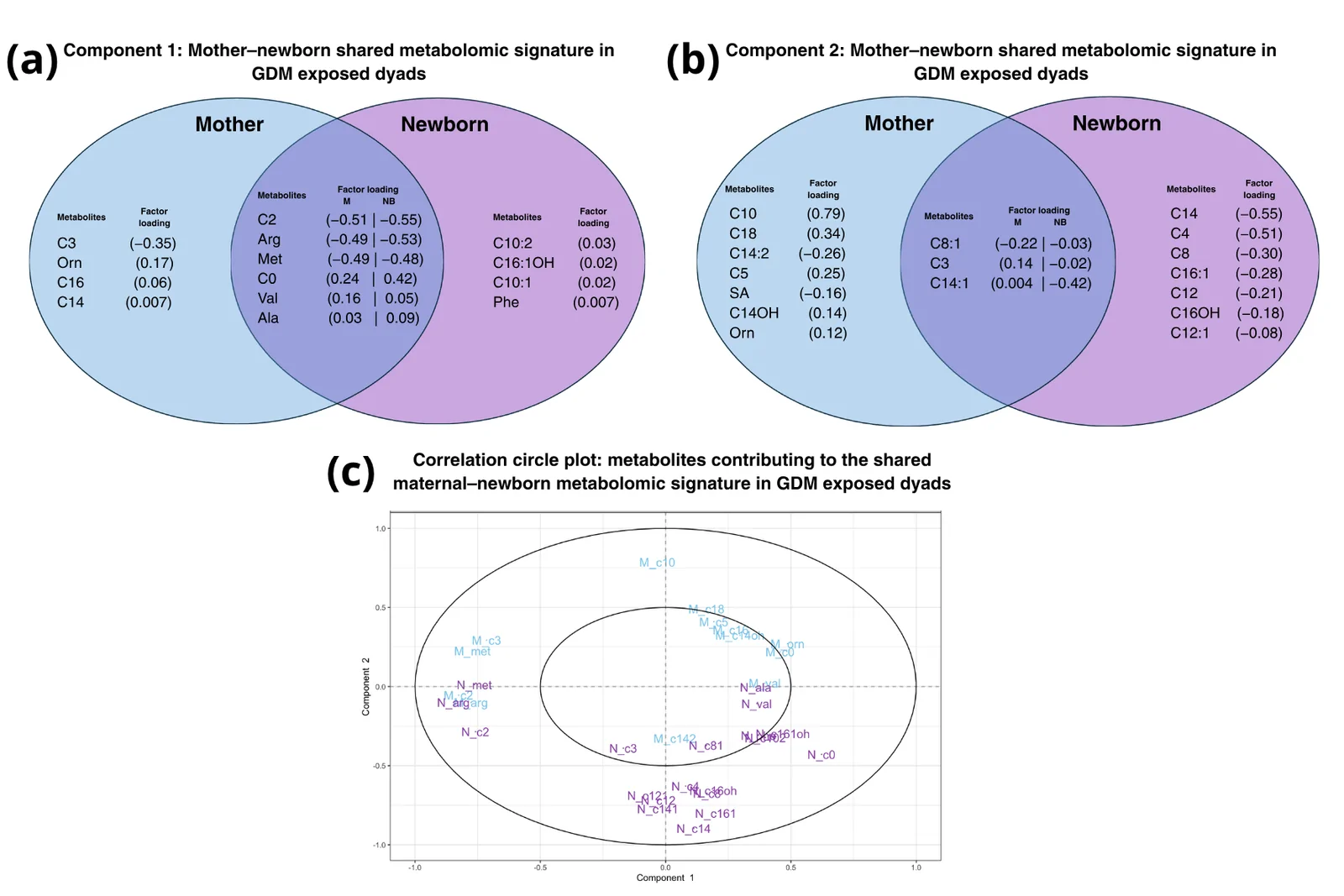
Identification of the shared metabolomic signature in GDM-exposed mother–newborn dyads using sPLS. (**a**) Metabolites comprising the shared metabolomic signature of Component 1 in GDM mother–newborn dyads, together with their corresponding factor loadings. (**b**) Metabolites comprising the shared metabolomic signature of Component 2 in GDM mother–newborn dyads, together with their corresponding factor loadings. (**c**) Correlation circle showing the association of maternal and neonatal metabolites with Components 1 and 2 in GDM mother–newborn dyads. Purple (N) indicates newborn metabolites, whereas blue (M) indicates maternal metabolites.

Stratified sPLS analysis showed that the amino acid and short-chain acylcarnitine signature identified in Component 1 was highly similar in GDM and non-GDM dyads (r = 0.908 and r = 0.909, respectively). Arginine, methionine, C2, and C0 constituted the core shared metabolites in both groups (Figure 2a and Figure 3a). In addition, valine and alanine contributed to the shared metabolomic signature exclusively in GDM dyads, suggesting that GDM may modify the maternal–newborn metabolic co-structure (Figure 3a).

In contrast, Component 2 showed a different metabolomic co-structure between GDM and non-GDM dyads, although in both cases this component was predominantly defined by acylcarnitines. In non-GDM dyads (r = 0.517), the shared metabolites were C18:1, C0 and octadecanoylcarnitine (C18), all with concordant loading directions, suggesting coordinated maternal–neonatal lipid transfer.

In GDM dyads (r = 0.687), the shared metabolites were octenoylcarnitine (C8:1), C3, and tetradecenoylcarnitine (C14:1); however, the latter two metabolites showed opposing loading directions between maternal and neonatal blocks, indicating an inverse maternal–neonatal association (Figure 3b).

### 2.4. Association Between GDM Status and Maternal–Neonatal Relationships of Metabolites Contributing to the Shared Signatures

To further characterize maternal–neonatal metabolic relationships, we calculated newborn-to-maternal (N/M) concentration ratios for all measured metabolites. Table 2 presents the N/M concentration ratios of metabolites contributing to the shared metabolomic signatures, stratified by GDM status. N/M concentration ratios for all measured metabolites are provided in Supplementary Table S2. After adjustment for multiple comparisons using the Benjamini–Hochberg False Discovery Rate (FDR) procedure, only the N/M ratio for valine differed significantly between groups. The median N/M ratios for valine were below 1 in both groups, indicating lower neonatal concentrations relative to maternal concentrations. However, the median ratio was lower in GDM dyads than in non-GDM dyads (0.685 vs. 0.809, respectively; FDR-adjusted *p* = 0.0097).

**Figure 4:**
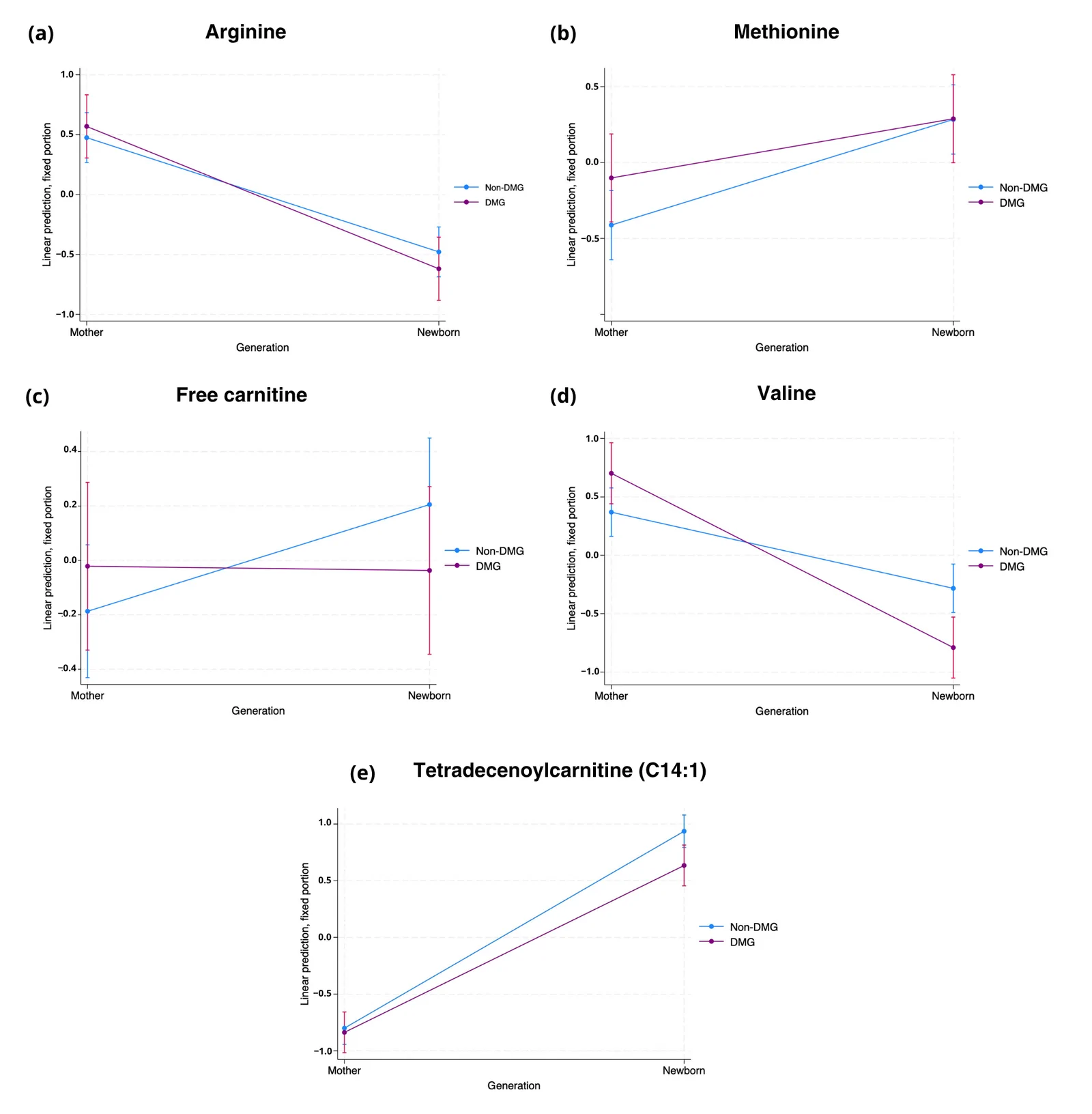
Adjusted estimated marginal means and 95% confidence intervals (CIs) for arginine, methionine, free carnitine, and valine. (**a**) Adjusted estimated marginal mean for arginine. (**b**) Adjusted estimated marginal mean for methionine. (**c**) Adjusted estimated marginal mean for free carnitine. (**d**) Adjusted estimated marginal mean for valine. (**e**) Adjusted estimated marginal mean for tetradecenoylcarnitine (C14:1). Marginal means were adjusted by maternal age, pre-pregnancy BMI, gestational age at birth and newborn sex.

Subsequently, we fitted linear mixed-effects models to evaluate whether GDM modified maternal–neonatal differences in the concentrations of individual metabolites contributing to the shared metabolomic signatures (Table 3). The newborn-by-GDM interaction was significant for arginine (β = −0.235, *p* = 0.068), methionine (β = −0.306, *p* = 0.033), free carnitine (β = −0.408, *p* = 0.062), valine (β = −0.840, *p* < 0.001), C14:1 (β = −0.265, *p* = 0.096), indicating that GDM modified the maternal–newborn relationship for these metabolites. Estimated marginal means showed that GDM-exposed dyads exhibited a more pronounced maternal-to-newborn decrease in arginine and valine, an attenuated increase in methionine and C14:1, and no increase in free carnitine, in contrast to the increase observed in non-GDM-exposed dyads (Figure 4a–e). Consistent with the findings from the N/M concentration ratio analysis, a significant difference in adjusted marginal mean concentrations between GDM-exposed and non-GDM-exposed newborns was observed only for valine, with lower log-transformed and standardized concentrations in GDM-exposed newborns (−0.788 vs. −0.284, respectively; *p* = 0.004; Figure 4d). Marginal means were adjusted for maternal age, pre-pregnancy BMI, gestational age at birth, and newborn sex.

## 3. Discussion

Our results demonstrate a robust presence of a core shared metabolomic signature between mothers and their newborns, composed of arginine, methionine, C2, and C0, which was consistently identified regardless of GDM exposure. However, in GDM mother–newborn dyads, valine and alanine additionally contributed to the shared metabolomic signature, suggesting that GDM partially modifies the shared metabolic relationship between mothers and their newborns. Additionally, a less robust signature comprising C8:1, C3, and C14:1 was identified specifically in GDM dyads.

Furthermore, mixed-effects models suggested that the effect of GDM on individual metabolites differed between mothers and newborns, indicating that although the core shared metabolomic signature was preserved, GDM differentially influenced specific metabolites within the mother–newborn dyad. Additionally, the analysis of N/M concentration ratios provided complementary evidence regarding the relative distribution of metabolites within mother–newborn dyads. Although several metabolites showed differences in their N/M ratios according to GDM status, only valine remained statistically significant after correction for multiple comparisons. Together, these findings provide evidence of the close metabolic integration between mothers and their newborns and offer further insight into the metabolic mechanisms underlying early metabolic programming.

The metabolites comprising the core shared metabolomic signature identified in mother–newborn dyads, regardless of GDM exposure, play key roles in fetal growth and development, supporting the biological relevance of the shared metabolic relationship observed between mothers and their newborns. Arginine is an amino acid involved in the regulation of placental blood flow, trophoblast growth, nutrient sensing, and organogenesis [9]. It is the precursor of nitric oxide (NO), which plays a critical role in trophoblast invasion, vascular function, and placental development [10]. In addition, arginine serves as a precursor for polyamines, which are essential for fertilization, implantation, embryonic development, placental angiogenesis, and fetal growth [9,11]. When arginine was analyzed individually, adjusted marginal mean concentrations did not differ significantly between mothers with and without GDM or between GDM-exposed and non-exposed newborns, despite a significant mother–newborn-by-GDM interaction. In mothers, our findings are consistent with a previous meta-analysis [12] showing no association between arginine concentrations at delivery and GDM. In newborns, evidence based on neonatal blood samples remains limited, as most studies have been conducted using cord blood. In contrast to our findings, a study conducted in a Chinese population reported lower arginine concentrations in neonatal blood among GDM-exposed newborns [13]; however, another study, consistent with our findings, found no association between neonatal arginine concentrations and GDM exposure [14].

In turn, methionine is closely linked to developmental programming because of its central role in one-carbon metabolism. As the precursor of S-adenosylmethionine (SAM), the universal methyl donor, methionine is essential for DNA and histone methylation, thereby regulating gene expression. Through these mechanisms, methionine contributes to epigenetic programming, which regulates multiple developmental processes, from blastocyst formation and development to cell differentiation, organogenesis, and fetal growth [15]. In our study, methionine was part of the shared metabolomic signature in mother–newborn dyads regardless of GDM status, underscoring the close relation between mother and newborn metabolism. In the individual metabolite analysis, we found that GDM differentially affected methionine concentrations within the mother–newborn dyad. However, adjusted marginal mean concentrations did not differ significantly between GDM-exposed and non-exposed groups within either mothers or newborns. Evidence regarding methionine concentrations in both mothers and newborns during the peripartum period is scarce.

C0 is a key regulator of energy metabolism. It is essential for mitochondrial fatty acid oxidation because it facilitates the transport of long-chain fatty acids across the inner mitochondrial membrane into the mitochondrial matrix, where they undergo β-oxidation [16]. The fetus has a limited capacity to synthesize carnitine because of the low activity of γ-butyrobetaine dioxygenase, the enzyme responsible for the final step of carnitine biosynthesis. Therefore, placental transfer from the mother represents the main source of fetal carnitine. At the end of pregnancy, maternal–fetal carnitine transfer increases, allowing carnitine to accumulate in fetal tissues and provide a reserve for the immediate postnatal period [17,18,19]. Similar to the findings for arginine and methionine, the maternal–newborn pattern of C0 concentrations differed according to GDM status, although adjusted marginal mean concentrations did not differ significantly between GDM-exposed and non-exposed groups within either mothers or newborns. Previous evidence regarding C0 concentrations in GDM remains limited and inconsistent in both mothers and newborns [14,20,21].

C2 is the most abundant acylcarnitine in human plasma [16] and plays a central role in energy metabolism by buffering the mitochondrial acetyl-CoA/CoA ratio through the reversible transfer of acetyl groups between acetyl-CoA and carnitine. This mechanism supports metabolic flexibility by facilitating the transition between glucose oxidation during feeding and fatty acid oxidation during fasting [18]. Although acetylcarnitine was part of the shared metabolomic signature independently of GDM status, when analyzed individually, its concentrations were not significantly associated with GDM status, nor did the maternal–newborn pattern of C2 concentrations differ according to GDM status.

In mother–newborn dyads, the contribution of carnitine and C0 to the shared metabolomic signature may reflect the integration of maternal carnitine availability, placental metabolism, and fetal energy metabolism during development and the metabolic adaptation to extrauterine life.

The involvement of these metabolites in interconnected metabolic pathways provides biological plausibility for their identification as a shared metabolomic signature in mother–newborn dyads, regardless of GDM exposure. Arginine and methionine are involved in processes related to uteroplacental blood flow, placental development, organogenesis, and epigenetic programming, while C0 and C2 play central roles in energy metabolism, mitochondrial function, and metabolic adaptation. Our findings suggest that, although arginine, methionine, and free carnitine play a central roles in mother–newborn metabolic interplay, GDM may alter the metabolic relationship between the maternal and neonatal compartments.

In mother–newborn dyads exposed to GDM, valine and alanine also contributed to the shared metabolomic signature. Valine is a branched-chain amino acid (BCAA) that has been associated with insulin resistance, T2DM [22] and cardiovascular diseases [23] in non-pregnant populations. Although we did not observe significant differences in adjusted marginal mean valine concentrations between mothers with and without GDM, previous studies have reported higher circulating valine concentrations in pregnant women with GDM compared with those without GDM [24,25,26]. In contrast, marginal means of valine concentrations in newborns exposed to GDM were lower than in non-exposed newborns. Studies evaluating postnatal blood samples from newborns are limited and have generally found no association between valine concentrations and GDM status [14]. Similarly, findings from cord blood studies have been limited and inconsistent, with some reporting no significant differences in valine concentrations according to GDM status [27], whereas others have found lower concentrations in newborns exposed to GDM [28].

In turn, alanine is a glucogenic amino acid whose higher concentrations have been associated with insulin resistance and impaired glucose metabolism in non-pregnant populations [29,30]. Similarly, studies in pregnant women have reported positive associations between maternal alanine concentrations and the risk of GDM [31,32] and maternal hyperglycemia [33]. However, findings from cord blood samples have been less consistent, with some studies reporting lower concentrations of alanine in those exposed to GDM [34], while others have found higher concentrations [35]. Our findings suggest that alanine contributes to the shared metabolomic signature in mother–newborn dyads exposed to GDM; however, in contrast to valine, the mixed-effects models did not provide evidence that GDM differentially affected alanine concentrations between mothers and newborns.

The shared metabolomic signature identified in Component 2 differed completely between GDM-exposed and non-exposed mother–newborn dyads. In non-exposed dyads, this signature was predominantly characterized by long-chain acylcarnitines, including C18 and C18:1, as well as C0. In contrast, in GDM-exposed dyads, the signature comprised acylcarnitines of different chain lengths, including the short-chain C3, medium-chain C8:1, and long-chain C14:1. Acylcarnitines and C0 concentrations may reflect fatty acid oxidation flux and mitochondrial substrate use. Accumulation of acylcarnitines, particularly when accompanied by reduced C0 availability, has been associated with incomplete fatty acid oxidation and mitochondrial dysfunction. More recently, acylcarnitines have been recognized as having roles beyond fatty acid oxidation, including epigenetic regulation, inflammation, immune homeostasis, endocrine regulation and neuromodulation. However, more evidence is needed to elucidate the specific biological functions of individual acylcarnitines [36]. Thus, the acylcarnitine signatures observed in GDM-exposed and non-exposed dyads may reflect differences in metabolic processes beyond fatty acid oxidation.

Despite significant differences in selected metabolic parameters between women with and without GDM, mothers with GDM did not exhibit marked alterations in conventional markers of metabolic control, which may reflect the clinical management received during pregnancy. Nevertheless, distinct shared metabolomic signatures were identified in GDM-exposed and non-exposed mother–newborn dyads. These findings suggest that metabolic alterations associated with GDM may persist beyond those captured by conventional clinical biomarkers, including glucose, glycated hemoglobin, and lipids. Moreover, they raise the possibility that additional metabolic pathways, not fully reflected by routine markers of glycemic and lipid control, may contribute to the maternal–newborn metabolic relationship and potentially to early metabolic programming.

Pharmacological management of GDM may have influenced our findings. Previous studies analyzing maternal [37] and umbilical cord blood [38] samples have reported higher concentrations of alanine in women treated with metformin and their newborns compared with those receiving insulin or dietary management. Moreover, experimental studies have shown that insulin increases the activity of the placental system A amino acid transporters [39], whereas metformin decreases the expression of large neutral amino acid transporters (LAT3 and LAT4) in trophoblast cells [40]. However, whether these effects translate into changes in fetal nutrient exposure under clinical treatment conditions remains uncertain. Considering the small sample sizes across treatment groups in our study, we were unable to reliably assess the potential contribution of pharmacological treatment to the observed metabolomic profiles.

To our knowledge, this is the first study to identify a shared metabolomic signature in mother–newborn dyads and to evaluate whether GDM status modifies this signature using an integrative multivariate model. Our findings provide new insights into maternal–newborn metabolic relationships and their potential role in early metabolic programming, which deserve further investigation in larger studies including a broader panel of metabolites.

When interpreting our results, some limitations should be considered. First, our study included a non-probability sample recruited from a single study center, which may limit the generalizability of our findings. Second, the relatively small sample size may have affected the stability of the identified metabolomic signatures, particularly in the GDM-stratified analyses. Third, our metabolite panel was limited to amino acids and acylcarnitines, comprising only a fraction of the maternal–newborn metabolome. Finally, the cross-sectional design precludes establishing causal relationships.

Despite these limitations, our study has several strengths. The paired mother–newborn design allowed us to directly investigate metabolic relationships within the dyad. In addition, the use of an integrative multivariate approach enabled the identification of a shared metabolomic signature capturing the covariance between maternal and neonatal metabolic profiles. The complementary use of mixed-effects models further allowed us to characterize how GDM differentially affected individual metabolite concentrations across maternal and neonatal compartments. Furthermore, the use of newborn blood samples collected within the first 24 h of life, rather than cord blood, provided a unique opportunity to characterize the neonatal metabolic profile during the early transition to extrauterine life, when it may still reflect maternal metabolism and placental influences. This approach addresses an important gap in the literature, which has predominantly relied on cord blood samples.

## 4. Materials and Methods

### 4.1. Study Design and Population

This cross-sectional study was conducted at the Pediatric Obesity Clinic and Wellness Unit of the Hospital General de México “Dr. Eduardo Liceaga” and participants were recruited at the Department of Gynecology from March 2024 to March 2025. Mother–newborn dyads were consecutively recruited within 24 h of delivery. The Hospital General de México is a tertiary-care referral center that primarily serves a vulnerable population without access to social security.

Inclusion criteria were: (1) mother–newborn dyads with or without GDM exposure, and (2) maternal age ≥ 18 years. Exclusion criteria were: (1) preeclampsia or eclampsia; (2) pre-existing maternal systemic diseases, including neurological, thyroid, rheumatologic, renal, or hepatic disorders; (3) multiple gestation; and (4) obstetric or neonatal complications.

All participants provided written informed consent before enrollment. The study was approved by the Hospital’s Institutional Research, Ethics, and Biosafety for Human Research Committee (DI/23/303/05/24) and was conducted in accordance with the principles of the Declaration of Helsinki.

### 4.2. GDM Diagnosis

GDM was diagnosed between 24 and 28 weeks of gestation using a 75-g oral glucose tolerance test (OGTT). GDM was defined according to the International Association of Diabetes and Pregnancy Study Groups (IADPSG)/American Diabetes Association (ADA) criteria, whereby the diagnosis was established if one or more of the following plasma glucose thresholds were met or exceeded: fasting ≥ 92 mg/dL, 1-h ≥ 180 mg/dL, or 2-h ≥ 153 mg/dL (3). Information on GDM diagnosis was obtained from participants’ medical records.

### 4.3. Metabolomic Analyses

Dried blood spot samples were collected from mother–newborn dyads on filter paper within the first 24 h after birth. Targeted metabolomic profiling was performed using the NeoBase Non-derivatized Kit (PerkinElmer, Waltham, MA, USA) to quantify free carnitine, acylcarnitines (C2, C3, C4, C5, C5:1, C6, C8, C8:1, C10, C10:1, C10:2, C12, C12:1, C14, C14:1, C14:2, C14OH, C16, C16:1, C16:1OH, C16-OH, C18, C18:1, C18:1OH, C18:2, and C18OH), argininosuccinate (ASA), and 11 L-amino acids (alanine, glycine, arginine, methionine, proline, valine, leucine, phenylalanine, tyrosine, citrulline, and ornithine). Metabolite concentrations were determined by tandem mass spectrometry (MS/MS) using a Quattro Micro API mass spectrometer (MicroMass, Cary, NC, USA). All results were expressed in μM.

### 4.4. Sociodemographic, Anthropometric and Clinical Data

Maternal age, educational level, and marital status were obtained from medical records. Pre-pregnancy body mass index (pre-pregnancy BMI) was calculated using self-reported pre-pregnancy weight and measured height. Gestational weight gain was calculated as the difference between the last measured maternal weight before delivery and self-reported pre-pregnancy weight.

Information on gestational age at birth, mode of delivery, newborn sex, and anthropometric measurements, including birth weight and birth length, was obtained from medical records.

### 4.5. Statistical Analyses

To characterize the study population, means and standard deviations (SDs) were estimated for quantitative variables, while frequencies and percentages were used for categorical data. Differences between mother–newborn dyads without GDM (non-GDM-exposed dyads) and those with (GDM-exposed dyads) were assessed using Student’s *t*-test for continuous variables.

Metabolite concentrations were log-transformed and standardized (z-score transformation) prior to analysis. To identify shared metabolomic signatures within mother–newborn dyads, we performed an unsupervised sparse Partial Least Squares (sPLS) analysis using the canonical framework. The number of components was determined by cross-validated Q^2^ criteria (5-fold cross-validation, 50 repetitions), retaining components with Q^2^ > 0. The optimal number of variables per component was selected by maximizing the cross-validated correlation between maternal and neonatal latent scores. sPLS is a multivariate projection-based method that identifies pairs of latent components maximizing the covariance between two continuous datasets while simultaneously performing variable selection through LASSO penalization of the loading vectors. This approach identifies variables that contribute most to the shared covariance structure between the two datasets [41].

After identifying the shared metabolomic signatures in the overall sample, the sPLS analysis was repeated after stratifying mother–newborn dyads according to GDM status to assess whether this condition modified the shared metabolomic signatures between mothers and newborns. For stratified models, the same number of variables per component selected in the global model (keepX = 10, keepY = 10) was applied to each stratum to ensure comparability between GDM-exposed and non-exposed groups.

Finally, N/M concentration ratios were calculated for all measured metabolites to characterize relative metabolite concentrations within mother–newborn dyads. Scatter plots of paired maternal and neonatal metabolite concentrations, stratified by GDM status, were generated to visualize within-dyad relationships.

Subsequently, linear mixed-effects regression models with a random intercept for each mother–newborn dyad were fitted separately for each metabolite contributing to the shared metabolomic signatures identified in Components 1 and 2. The models included an interaction term between GDM status (non-GDM vs. GDM) and generation (mother vs. newborn) to assess whether maternal–neonatal differences in metabolite concentrations varied according to GDM status. Models were adjusted for maternal age, pre-pregnancy BMI, gestational age at birth, and newborn sex. Statistical analyses were performed using the mixOmics package in RStudio (version 2026.04.0+526; Posit Software, PBC, Boston, MA, USA) and Stata 19 (StataCorp, College Station, TX, USA). A *p*-value <0.05 was considered statistically significant, whereas a *p*-value < 0.10 was considered statistically significant for interaction terms.

## 5. Conclusions

Mother–newborn dyads showed a shared metabolomic signature composed of arginine, methionine, C0, and C2. This signature, comprising metabolites involved in placental and fetal growth and development, as well as energy metabolism, was conserved regardless of GDM status. However, the shared metabolomic signature of GDM-exposed mother–newborn dyads additionally included alanine and valine, suggesting that GDM may modify the metabolic relationship between mothers and newborns. Furthermore, GDM differentially affected the maternal–newborn concentration patterns of some individual metabolites. These findings provide insights into the metabolic processes that may contribute to fetal programming and support the value of studying the mother–newborn dyad as an integrated biological system. Longitudinal studies with larger sample sizes and broader metabolite panels are needed to better characterize metabolic interactions within the dyad and evaluate their association with cardiometabolic risk later in life.

## Figures and Tables

**Table 1:** Characteristics of mother–newborn dyads.

Variable	Total n = 112 Mean (SD)	Non-GDM n = 68 Mean (SD)	GDM n = 44 Mean (SD)	*p*-Value
**Maternal characteristics**				
Maternal age (years)	25.9 (6.3)	23.9 (5.2)	29.1 (6.5)	<0.001
Pre-pregnancy BMI (kg/m^2^)	26.1 (5.6)	24.6 (4.9)	28.5 (5.7)	<0.001
Gestational weight gain (kg)	9.9 (5.3)	10.1 (5.1)	9.5 (5.6)	0.449
Glucose (mg/dL)	72.4 (14.5)	70.5 (12.5)	75.3 (16.6)	0.014
Total cholesterol (mg/dL)	204.0 (42.7)	210.1 (33.6)	199.3 (43.3)	0.043
Triglycerides (mg/dL)	243.4 (71.0)	233.4 (69.8)	259.3 (69.9)	0.009
HDL cholesterol (mg/dL)	54.5 (12.8)	55.8 (12.6)	52.4 (13.0)	0.066
LDL cholesterol (mg/dL)	119.8 (35.3)	123.2 (32.3)	113.9 (39.3)	0.066
HbA1c (%)	5.5 (0.6)	5.4 (0.4)	5.6 (0.9)	0.022
**Neonatal characteristics**				
Gestational age at birth (weeks)	38.9 (1.5)	39.1 (1.4)	38.7 (1.6)	0.160
Birth weight (g)	3064 (402.0)	3030.9 (417.3)	3116.5 (375.4)	0.276
Birth length (cm)	49.3 (2.2)	49.6 (2.1)	48.9 (2.3)	0.082
Abdominal circumference (cm)	31.0 (2.4)	31.1 (2.4)	31.0 (2.4)	0.817
Head circumference (cm)	33.9 (1.6)	33.7 (1.5)	34.3 (1.6)	0.055
Mid-upper arm circumference (cm)	10.3 (1.0)	10.2 (1.1)	10.5 (1.0)	0.159

Abbreviations: Non-GDM, non-gestational diabetes exposed dyads; GDM, gestational diabetes dyads; SD, standard deviation; BMI, body mass index. Differences between groups were evaluated using Student’s *t*-test.

**Table 2:** Newborn-to-Maternal Concentration Ratios of Metabolites Contributing to the Shared Metabolomic Signatures in Non-GDM and GDM Dyads.

Metabolites	N/M Ratio Non-GDM Dyads	N/M Ratio GDM Dyads	Unadjusted *p*-Value	FDR-Adjusted *p*-Value
**Amino acids**				
Arginine	0.463 (0.347–0.617)	0.439 (0.259–0.559)	0.200	0.512
Alanine	0.883 (0.711–1.111)	0.896 (0.766–1.265)	0.425	0.604
Methionine	1.389 (1.129–1.817)	1.170 (0.959–1.539)	0.029 *	0.224
Valine	0.809 (0.683–0.985)	0.685 (0.575–0.766)	<0.0001 *	0.009 **
**Acylcarnitines**				
C0	1.116 (0.911–1.401)	0.903 (0.792–1.248)	0.052	0.242
C2	2.007 (1.545–2.936)	1.815 (1.211–2.677)	0.238	0.516
C3	1.606 (1.232–2.070)	1.514 (1.111–1.937)	0.223	0.512
C8:1	1.160 (0.929–1.348)	1.077 (0.931–1.297)	0.995	0.995
C14:1	2.000 (1.625–2.438)	1.866 (1.410–2.310)	0.125	0.374
C18	2.850 (2.298–3.510)	2.908 (2.232–3.547)	0.832	0.877
C18:1	1.424 (1.178–1.804)	1.410 (1.126–1.593)	0.312	0.554

Non-GDM, without gestational diabetes mellitus; GDM, gestational diabetes mellitus; N/M ratio, newborn/maternal ratio. Data are presented as median (25th–75th percentile). Unadjusted *p*-values were calculated using the Mann–Whitney U test to compare N/M concentration ratios between the non-GDM and GDM groups. *p*-values were subsequently adjusted for multiple comparisons using the Benjamini–Hochberg false discovery rate (FDR) procedure. * Statistically significant unadjusted *p*-values (*p* < 0.05). ** Statistically significant FDR-adjusted *p*-values (*p* < 0.05).

**Table 3:** Interaction Between Generation (Mother vs. Newborn) and GDM Status (Non-GDM vs. GDM) for Metabolites Comprising the Shared Metabolomic Signatures.

Metabolite	β (Newborn × GDM)	95% CI	*p*-Value
Arginine ^†^	−0.235	−0.487 to 0.017	0.068 *
Methionine ^†^	−0.306	−0.587 to −0.025	0.033 *
Acetylcarnitine (C2) ^†^	−0.191	−0.450 to 0.068	0.149
Free carnitine (C0) ^†^	−0.408	−0.836 to 0.021	0.062 *
Propionylcarnitine (C3) ^‡, #^	−0.177	−0.504 to 0.151	0.290
Valine ^§^	−0.84	−1.289 to −0.392	<0.001 *
Alanine ^§^	0.142	−0.350 to 0.635	0.571
Oleoylcarnitine (C18:1) ^¶^	−0.263	−0.649 to 0.122	0.180
Octadecanoylcarnitine (C:18) ^¶^	−0.002	−0.225 to 0.221	0.984
Octenoylcarnitine (C8:1) ^#^	−0.013	−0.471 to 0.445	0.955
Tetradecenoylcarnitine (C14:1) ^#^	−0.265	−0.576 to 0.047	0.096 *

Models adjusted by maternal age, pre-pregnancy BMI, gestational age at birth and newborn sex. * Statistically significant at *p* < 0.10. ^†^ Metabolites contributing to the shared metabolomic signature of Component 1 in both non-GDM and GDM dyads. ^‡^ Metabolites contributing to the shared metabolomic signature of Component 1 in non-GMD dyads. ^§^ Metabolites contributing to the shared metabolomic signature of Component 1 in GMD dyads. ^¶^ Metabolites contributing to the shared metabolomic signature of Component 2 in non-GMD dyads. ^#^ Metabolites contributing to the shared metabolomic signature of Component 2 in GMD dyads.

## Data Availability

The data presented in this study are available on request from the corresponding author.

## Supplementary Materials

The following supporting information can be downloaded at https://www.mdpi.com/article/10.3390/ijms27219786/s1.

## Abbreviations

The following abbreviations are used in this manuscript:

GDM	gestational diabetes
T2DM	type 2 diabetes mellitus
BMI	body mass index
SD	standard deviation
sPLS	sparse partial least squares
N/M	newborn-to-maternal
FDR	false discovery rate
OGTT	oral glucose tolerance test
C0	free carnitine
C2	acetylcarnitine
C3	propionylcarnitine
C4	butyrylcarnitine
C6	hexanoylcarnitine
C8:1	octenoylcarnitine
C12:1	dodecenoylcarnitine
C14:1	tetradecenoylcarnitine
C16-OH	3-hydroxyhexadecanoylcarnitine
C18:1	octadecenoylcarnitine
C18	octadecanoylcarnitine
