# Supplementary material for: Identifying a Shared Metabolomic Signature in Mother–Newborn Dyads With and Without Gestational Diabetes: Implications for Early Metabolic Programming
Source: Int J Mol Sci. 2026 Oct 31;27(21):9786. doi: 10.3390/ijms27219786 (PMC13763059; doi:10.3390/ijms27219786)
Supplement: Supplementary file 1 [file ijms-27-09786-s001.zip › Supplementary figure 1.pdf]

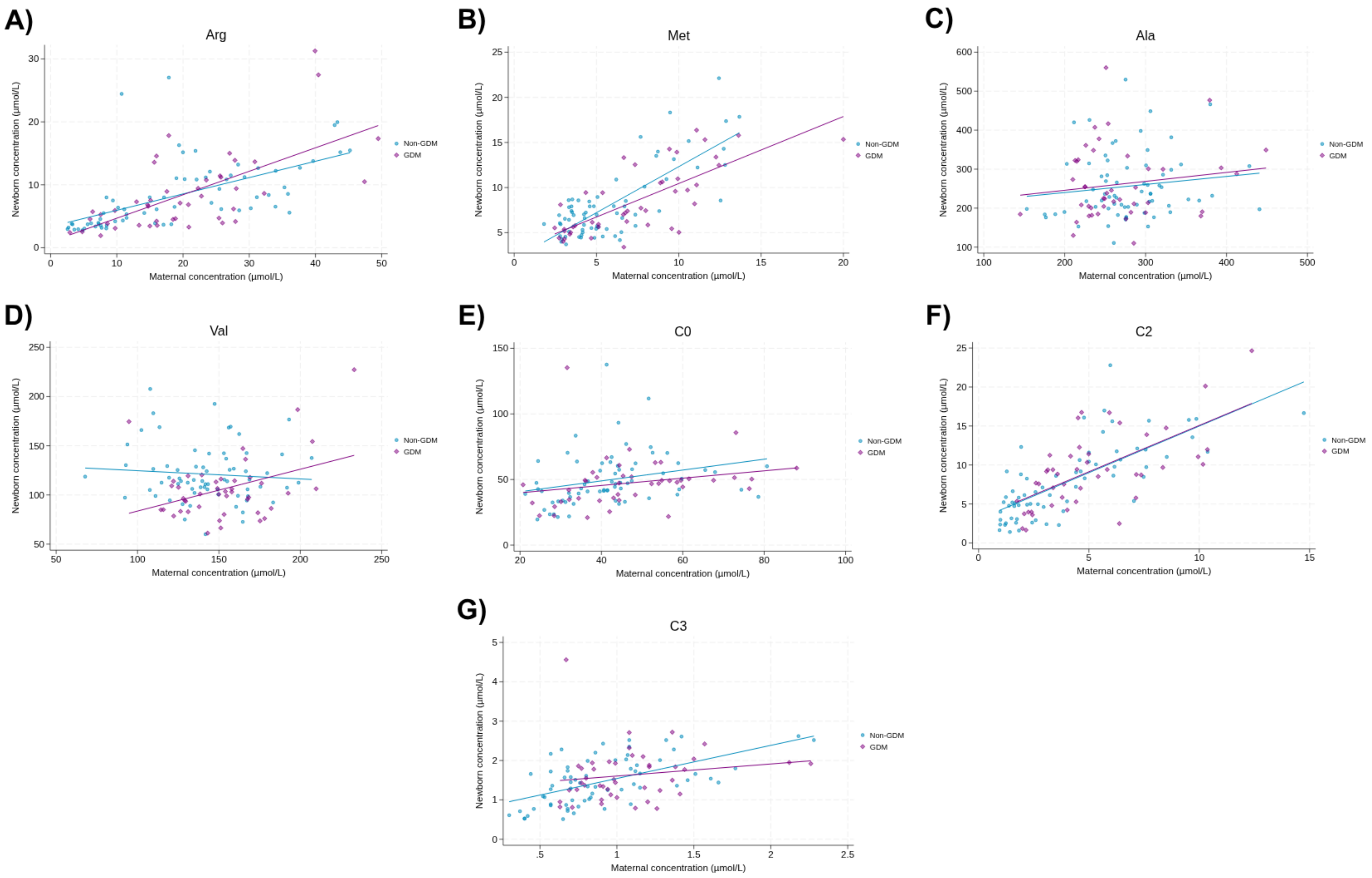

Supplementary figure S1. Scatter plots of paired maternal and neonatal concentrations of metabolites contributing to the shared metabolomic signatures in Component 1, stra tified by GDM status. A) Arginine, B) methionine, C) alanine, D) valine, E) free carnitine (C0), F) acetylcarnitine (C2), and G) propionylcarnitine (C3). Each point represents an individual mo ther–newborn dyad. Solid lines represent group-specific linear regression fits. Metabolite concentrations are expressed in µmol/L.
