# Supplementary material for: Identifying a Shared Metabolomic Signature in Mother–Newborn Dyads With and Without Gestational Diabetes: Implications for Early Metabolic Programming
Source: Int J Mol Sci. 2026 Oct 31;27(21):9786. doi: 10.3390/ijms27219786 (PMC13763059; doi:10.3390/ijms27219786)
Supplement: Supplementary file 1 [file ijms-27-09786-s001.zip › Table S1.pdf]

Table S1. Absolute metabolite concentrations in mothers and newborns according to GDM status.

Metabolite	Non-GDM mothers n=68	GDM mothers n=44	Unadjusted p-value	FDR-adjusted p-value	Non-GDM newborns n=68	GDM newborns n=44	Unadjusted p-value	FDR-adjusted p-value
**Amino acids**		data						
Arginine	17.50 (8.21–28.40)	18.68 (14.01–26.50)	0.464	0.723	6.43 (3.85–11.20)	6.83 (4.20–10.97)	0.910	0.934
Citrulline	20.71 (17.50–24.91)	20.96 (17.22–22.88)	0.411	0.697	21.41 (19.12–26.00)	21.20 (17.92–26.04)	0.292	0.632
Glycine	289.62 (253.61–342.98)	297.93 (258.62–358.39)	0.575	0.775	507.71 (442.66–566.53)	529.79 (451.67–603.82)	0.194	0.582
Alanine	275.29 (250.69–306.07)	250.23 (227.90–286.65)	0.044*	0.215	231.84 (198.44–305.70)	231.84 (190.03–321.27)	0.896	0.934
Leucine	115.46 (100.25–126.89)	118.80 (110.58–133.69)	0.058	0.227	90.70 (82.80–105.69)	86.53 (77.36–95.72)	0.144	0.467
Methionine	4.93 (3.52–7.37)	6.86 (4.03–9.91)	0.036*	0.202	7.01 (5.42–8.65)	7.42 (5.49–10.80)	0.547	0.762
Phenylalanine	45.87 (43.32–49.55)	48.63 (41.73–51.95)	0.229	0.446	57.59 (50.28–69.19)	51.17 (43.85–58.84)	0.023*	0.226
Tyrosine	41.86 (36.06–47.68)	45.03 (40.69–52.61)	0.025*	0.199	97.72 (78.46–126.45)	96.59 (78.43–111.97)	0.905	0.934
Valine	142.11 (125.34–162.06)	151.21 (130.30–168.71)	0.099	0.250	114.63 (105.25–133.60)	101.35 (84.98–114.17)	<0.001*	0.034**
Ornithine	59.80 (48.20–74.86)	58.90 (52.70–67.97)	0.938	0.963	86.07 (74.83–105.65)	79.17 (68.11–114.73)	0.453	0.705
Proline	118.21 (106.57–133.90)	114.97 (102.89–133.76)	0.482	0.723	175.45 (153.61–203.78)	165.41 (140.74–189.04)	0.124	0.467
**Acylcarnitines**								
C0	41.835 (32.185–47.490)	44.375 (36.080–56.615)	0.096	0.250	45.340 (37.635–60.270)	47.585 (35.175–51.900)	0.703	0.857
C2	3.155 (1.720–6.015)	4.485 (3.185–6.765)	0.013*	0.177	6.880 (4.675–10.855)	9.005 (5.565–11.330)	0.133	0.467
C3	0.810 (0.655–1.100)	0.990 (0.820–1.235)	0.005*	0.177	1.435 (0.985–1.855)	1.510 (1.195–1.935)	0.282	0.632
C4	0.130 (0.120–0.160)	0.155 (0.130–0.180)	0.012*	0.177	0.220 (0.190–0.280)	0.225 (0.205–0.265)	0.365	0.648
C5	0.095 (0.080–0.110)	0.100 (0.090–0.120)	0.186	0.403	0.160 (0.130–0.200)	0.160 (0.140–0.205)	0.693	0.857
C5:1	0.045 (0.040–0.060)	0.050 (0.040–0.060)	0.107	0.250	0.080 (0.060–0.090)	0.070 (0.060–0.090)	0.261	0.632
C6	0.050 (0.040–0.050)	0.050 (0.040–0.060)	0.105	0.250	0.070 (0.070–0.085)	0.080 (0.060–0.090)	0.767	0.879
C8	0.060 (0.050–0.070)	0.060 (0.050–0.080)	0.058	0.227	0.100 (0.080–0.110)	0.090 (0.080–0.105)	0.364	0.648
C8:1	0.130 (0.110–0.150)	0.140 (0.130–0.150)	0.109	0.250	0.150 (0.130–0.170)	0.160 (0.130–0.180)	0.358	0.648
C10	0.110 (0.100–0.130)	0.120 (0.090–0.135)	0.288	0.535	0.140 (0.120–0.170)	0.140 (0.120–0.160)	0.967	0.934
C10:1	0.160 (0.145–0.180)	0.170 (0.160–0.180)	0.068	0.243	0.180 (0.170–0.205)	0.170 (0.160–0.195)	0.253	0.632
C10:2	0.040 (0.030–0.050)	0.040 (0.030–0.050)	0.632	0.795	0.060 (0.050–0.080)	0.060 (0.045–0.080)	0.399	0.650
C12	0.060 (0.050–0.070)	0.060 (0.050–0.070)	0.793	0.883	0.140 (0.120–0.170)	0.130 (0.100–0.160)	0.020*	0.226
C12:1	0.115 (0.100–0.130)	0.120 (0.110–0.130)	0.031*	0.199	0.210 (0.180–0.240)	0.195 (0.170–0.225)	0.239	0.632
C14	0.060 (0.050–0.070)	0.060 (0.050–0.070)	0.592	0.775	0.200 (0.175–0.240)	0.200 (0.160–0.240)	0.400	0.650
C14:1	0.080 (0.070–0.080)	0.080 (0.070–0.090)	0.975	0.975	0.155 (0.125–0.180)	0.140 (0.120–0.170)	0.073	0.467
C14:2	0.030 (0.030–0.040)	0.030 (0.030–0.040)	0.596	0.775	0.050 (0.040–0.060)	0.050 (0.040–0.055)	0.844	0.934
C14-OH	0.020 (0.020–0.030)	0.020 (0.020–0.020)	0.382	0.678	0.040 (0.030–0.050)	0.035 (0.030–0.045)	0.117	0.467
C16	0.570 (0.525–0.670)	0.595 (0.510–0.725)	0.581	0.775	2.505 (2.105–3.245)	2.505 (2.180–3.140)	0.766	0.879

C16:1	0.070 (0.060–0.080)	0.070 (0.065–0.080)	0.457	0.723	0.275 (0.225–0.335)	0.265 (0.215–0.325)	0.331	0.648
C16:1-OH	0.040 (0.030–0.050)	0.040 (0.030–0.050)	0.829	0.898	0.070 (0.060–0.085)	0.070 (0.060–0.080)	0.120	0.467
C16-OH	0.035 (0.030–0.040)	0.030 (0.030–0.040)	0.213	0.438	0.060 (0.050–0.060)	0.050 (0.040–0.060)	0.013*	0.226
C18	0.240 (0.210–0.290)	0.235 (0.215–0.275)	0.926	0.963	0.690 (0.570–0.840)	0.720 (0.595–0.815)	0.533	0.762
C18:1	0.580 (0.540–0.695)	0.665 (0.560–0.785)	0.030*	0.199	0.895 (0.740–1.090)	0.905 (0.765–1.090)	0.666	0.857
C18:1-OH	0.030 (0.020–0.030)	0.030 (0.020–0.030)	0.780	0.883	0.050 (0.040–0.060)	0.040 (0.040–0.050)	0.119	0.467
C18:2	0.255 (0.225–0.290)	0.275 (0.215–0.295)	0.779	0.883	0.180 (0.145–0.225)	0.190 (0.150–0.240)	0.470	0.705
C18-OH	0.020 (0.010–0.020)	0.020 (0.020–0.020)	0.100	0.250	0.040 (0.030–0.040)	0.030 (0.030–0.040)	0.112	0.467
**Other metabolites**								
Arginosuccinate	2.75 (2.59–2.84)	2.74 (2.60–2.87)	0.779	0.883	2.90 (2.81–3.06)	2.90 (2.78–3.06)	0.668	0.857

Non-GDM, without gestational diabetes mellitus; GDM, gestational diabetes mellitus. Data are presented as median (25th–75th percentile). Unadjusted p-values were calculated using the Mann–Whitney U test to compare N/M concentration ratios between the non-GDM and GDM groups. P-values were subsequently adjusted for multiple comparisons using the Benjamini–Hochberg false discovery rate (FDR) procedure.

* Statistically significant unadjusted p-values ($p < 0.05$).

** Statistically significant FDR-adjusted p-values ($p < 0.05$).
