# Supplementary material for: Identifying a Shared Metabolomic Signature in Mother–Newborn Dyads With and Without Gestational Diabetes: Implications for Early Metabolic Programming
Source: Int J Mol Sci. 2026 Oct 31;27(21):9786. doi: 10.3390/ijms27219786 (PMC13763059; doi:10.3390/ijms27219786)
Supplement: Supplementary file 1 [file ijms-27-09786-s001.zip › Table S2.pdf]

Table S1. Newborn-to-maternal metabolite concentration ratios in mother–newborn dyads with and without GDM

Metabolite	N/M ratio non-GDM dyads	N/M ratio GDM dyads	Unadjusted p-value	FDR-adjusted p-value
**Amino acids**				
Arginine	0.463 (0.347–0.617)	0.439 (0.259–0.559)	0.200	0.512
Citrulline	1.117 (0.875–1.328)	1.064 (0.892–1.391)	0.967	0.992
Glycine	1.669 (1.359–2.132)	1.730 (1.440–2.285)	0.539	0.637
Alanine	0.883 (0.711–1.111)	0.896 (0.766–1.265)	0.425	0.604
Leucine	0.798 (0.685–1.046)	0.720 (0.603–0.824)	0.011*	0.206
Methionine	1.389 (1.129–1.817)	1.170 (0.959–1.539)	0.029*	0.224
Phenylalanine	1.261 (1.016–1.485)	1.102 (0.924–1.340)	0.021*	0.206
Tyrosine	2.378 (1.738–2.989)	2.123 (1.709–2.728)	0.213	0.512
Valine	0.809 (0.683–0.985)	0.685 (0.575–0.766)	<0.0001*	<0.001**
Ornithine	1.436 (1.190–1.773)	1.384 (1.123–1.789)	0.825	0.877
Proline	1.496 (1.235–1.828)	1.384 (1.169–1.719)	0.362	0.588
**Acylcarnitines**				
C0	1.116 (0.911–1.401)	0.903 (0.792–1.248)	0.052	0.242
C2	2.007 (1.545–2.936)	1.815 (1.211–2.677)	0.238	0.516
C3	1.606 (1.232–2.070)	1.514 (1.111–1.937)	0.223	0.512
C4	1.645 (1.387–2.000)	1.433 (1.236–1.878)	0.083	0.294
C5	1.784 (1.422–2.100)	1.633 (1.400–2.095)	0.631	0.724
C5:1	1.667 (1.310–2.200)	1.500 (1.000–1.817)	0.049	0.242
C6	1.600 (1.333–2.000)	1.600 (1.200–1.929)	0.429	0.604
C8	1.500 (1.375–1.833)	1.429 (1.000–1.833)	0.096	0.314
C8:1	1.160 (0.929–1.348)	1.077 (0.931–1.297)	0.995	0.995
C10	4.214 (3.404–5.335)	1.231 (1.000–1.606)	0.266	0.546
C10:1	4.134 (2.944–4.917)	1.059 (0.943–1.214)	0.062	0.242
C10:2	1.750 (1.500–2.333)	1.367 (1.000–2.000)	0.282	0.551
C12	1.550 (1.333–2.000)	2.155 (1.757–3.000)	0.057	0.242
C12:1	1.273 (1.074–1.634)	1.583 (1.360–1.909)	0.048	0.242
C14	1.167 (0.943–1.371)	3.071 (2.500–4.083)	0.412	0.604
C14:1	1.500 (1.225–2.000)	1.866 (1.410–2.310)	0.125	0.374
C14:2	2.633 (2.000–3.367)	1.583 (1.250–2.000)	0.669	0.746
C14-OH	1.809 (1.442–2.215)	1.583 (1.333–2.500)	0.509	0.637
C16	3.400 (2.714–3.900)	4.109 (3.260–5.209)	0.444	0.604
C16:1	2.000 (1.625–2.438)	3.500 (2.690–4.429)	0.145	0.404
C16:1-OH	1.500 (1.000–1.833)	1.775 (1.367–2.208)	0.477	0.620
C16-OH	2.000 (1.500–2.500)	1.500 (1.333–1.750)	0.302	0.554
C18	2.850 (2.298–3.510)	2.908 (2.232–3.547)	0.832	0.877
C18:1	1.424 (1.178–1.804)	1.410 (1.126–1.593)	0.312	0.554
C18:1-OH	1.667 (1.333–2.000)	1.667 (1.333–2.000)	0.538	0.637
C18:2	0.686 (0.574–0.852)	0.768 (0.574–0.911)	0.449	0.604
C18-OH	2.000 (1.500–3.000)	1.500 (1.500–2.500)	0.017*	0.206
**Other metabolites**				
Arginosuccinate	1.080 (1.020–1.140)	1.057 (1.009–1.122)	0.343	0.582

Non-GDM, without gestational diabetes mellitus; GDM, gestational diabetes mellitus; N/M ratio, newborn/maternal ratio. Data are presented as median (25th–75th percentile). Unadjusted p-values were calculated using the Mann–Whitney U test to compare N/M concentration ratios between the non-GDM and GDM groups. P-values were subsequently adjusted for multiple comparisons using the Benjamini–Hochberg false discovery rate (FDR) procedure.

* Statistically significant unadjusted p-values ($p < 0.05$).

** Statistically significant FDR-adjusted p-values ($p < 0.05$).
